# mGem: Cut4/Apc1 and its prion form at the cross roads of cell cycle regulation, heterochromatin organization, RNAi, stress response, and evolution

**DOI:** 10.1128/mbio.00837-25

**Published:** 2026-03-30

**Authors:** Suman Sharma, Jagmohan Singh

**Affiliations:** 1 Amity University Punjab 657892, Sahibzada Ajit Singh Nagar, Punjab, India; Instituto Carlos Chagas, Curitiba, Brazil

**Keywords:** anaphase-promoting complex, epigenetics, heterochromatin, RNA interference, stress resppnse, prions

## Abstract

The anaphase-promoting complex/cyclosome (APC/C) complex plays a pivotal role in cell cycle progression in eukaryotes. APC/C-mediated polyubiquitination and subsequent degradation of regulatory factors ensure sister chromatid separation during mitosis and mitotic exit. Likewise, coordination between the chromodomain protein Swi6/HP1, histone methyltransferase Clr4/Suv39, and RNAi promotes heterochromatin assembly in *Schizosaccharomyces pombe*. Interestingly, Cut4/Apc1 and Cut9 subunits of APC/C regulate RNAi and interact with Swi6/HP1 and Clr4/Suv39 to promote mutual recruitment at heterochromatin. *sng2-1*, a mutant of Cut4/Apc1, can assume a prion form, designated [SNG2], with defective heterochromatin silencing. As in prions, this defect is inherited in a non-Mendelian manner, accompanied by aggregation of Cut4. Paradoxically, along with greater chromosome instability and aneuploidy—phenotypes associated with cancer, the prion form displayed enhanced stress tolerance, thereby providing a trade-off between defects in chromosome integrity and survival under stress conditions. Thus, the prion form may confer evolutionary advantage.

## PERSPECTIVE

**Fig 1:**
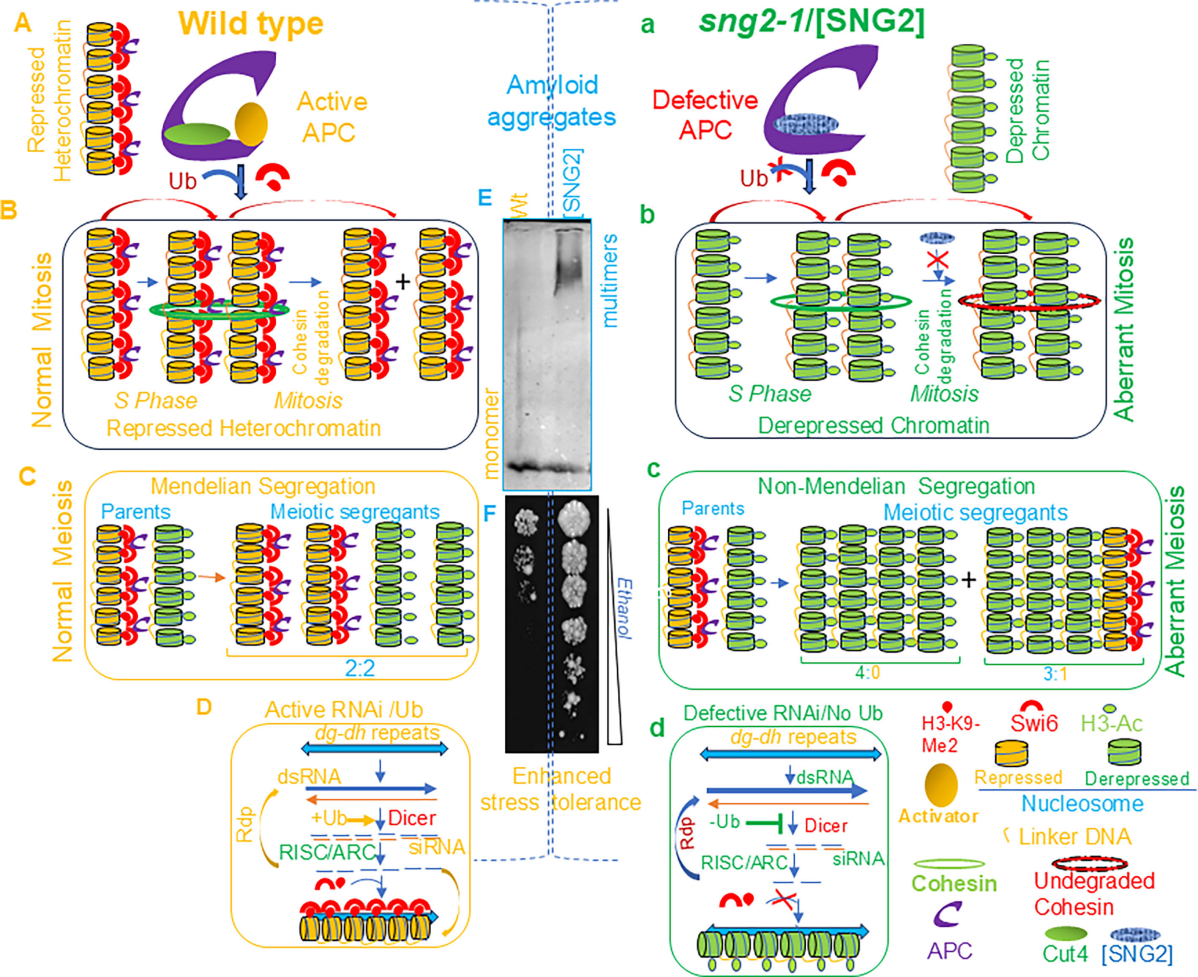
Interplay of normal and prion form of Cut4p with Swi6/HP1 and possible physiological outcomes. (Left) Role of APC/C in assembly of repressed heterochromatin by regulating the association of nucleosomes with Swi6/HP1 and Lys9-di-/tri-methylated H3 (H3-K9-Me2/3), shown in yellow (**A**), its integrity and equal transmission among daughter cells during mitosis due to regulated degradation of cohesin following ubiquitination by APC/C (indicated by arrow sign) (**B**), and inheritance as a Mendelian allele in a 2:2 ratio when crossed with a mutant strain having a derepressed state of heterochromatin (green) during meiosis (**C**). APC/C is also involved in the generation of siRNAs by modulating the activity of key RNAi factors through ubiquitination, which, in turn, may help in H3-Lys9-Me2/3 by Clr4/Suv39 and recruitment of Swi6/HP1, thereby the establishment of heterochromatin (+ Ub; yellow) (**D**). The repressed state of heterochromatin is represented in yellow color. (Right) In cells with the prion form [SNG2], lack of H3-K9-me caused by lack of recruitment of Clr4/Suv39 and Swi6/HP1 by [SNG2] leads to buildup of acetylated histone H3 (H3-Ac, green circle) (**a**), resulting in derepressed chromatin. The derepressed chromatin (shown in green) undergoes aberrant segregation during mitosis owing to blocked degradation of cohesin (red ring vs broken red ring; red X sign) (**b**) and is inherited in a non-Mendelian manner when [SNG2] cells (green) are crossed with a wild-type strain having a repressed state of chromatin (yellow) (**c**). The *sng2-1* mutant/[SNG2] prion, which lacks efficient ubiquitination (No Ub) and, therefore, fails to activate the specific RNAi factors, like Dicer, is defective in generation of siRNA from the *dg-dh* repeats and thereby defective in heterochromatin assembly (-Ub; green) (**d**) by Swi6/HP1 and H3-K9-me2/3 (red X sign) (**d**). The abovementioned phenotypes are caused by the formation of prion aggregates by Cut4p in the [SNG2] as compared to wild-type strains (wt), as visualized by SDDAGE analysis (center panel, **E**). Another consequence is that the [SNG2] cells display enhanced tolerance to ethanol stress, as indicated by growth assay on plates containing ethanol gradient (center panel, **F**).

An orderly cell cycle progression involves exact duplication of genetic material and its equal apportioning among daughter cells. Any defects in this process can lead to aneuploidy and diseases like cancer. This orderly progression is ensured by timely synthesis, functioning, and degradation of regulatory factors. APC/C plays a pivotal role in this pathway. It is a multisubunit 1.5 megadalton complex, an E3-ubiquitin ligase. Following its activation, APC/C polyubiquitinate-specific targets like securin and cyclin during cell cycle. Degradation of polyubiquitinated securin by the proteasome leads to degradation of the cohesin complex, which forms a rig around the sister chromatids during metaphase, triggering their separation of sister chromatids in anaphase (Fig. 1B). Likewise, degradation of polyubiquitinated cyclin by the proteasome triggers mitotic exit (1, 2).

A large component of eukaryotic genome is organized into heterochromatin. The centromere (*cen*) and telomere (*tel*) loci form constitutive heterochromatin. In fission yeast, the silent mating type (*mat*) loci are assembled into facultative heterochromatin (3). The structural integrity of heterochromatin is critical for chromosomal stability and equal transmission among daughter cells. The heterochromatin regions do not harbor any essential genes but are folded into an inaccessible higher-order structure characterized by transcriptional silencing of any reporter gene inserted therein. Several trans-acting factors, like Swi6, Clr1-Clr4, Polα, Polδ, Polε, Rhp6/Rad6, etc., are required for silencing. Mutations in these factors cause derepression of the heterochromatin loci as well as chromosome segregation defects (4–11).

Mechanistically, the heterochromatin structure is established and maintained by epigenetic mechanisms. The newly synthesized and hyperacetylated histones undergo deacetylation by the histone deacetylases, followed by methylation of histone H3 at the Lys9 position by the histone methyltransferase Clr4/Suv39. The Lys9-di/trimethylated histone H3 (H3-K9-me2/me3) is bound by Swi6/HP1 through its chromodomain to initiate heterochromatin assembly (yellow; Fig. 1A). A cooperative interaction between Swi6/HP1 and Clr4/Suv39 promotes heterochromatin spreading (12, 13). In contrast, the depressed state of heterochromatin is associated with acetylated histones (green; H3-Ac; Fig. 1a).

RNAi also plays a role in heterochromatin silencing in *Schizosaccharomyces pombe*. It acts through the *dg-dh* repeat sequences located at the *cen*, *mat*, and *tel* loci (14). Mutations in genes involved in RNAi, namely, *dcr1*, *rdp1*, and *ago1*, elicit loss of silencing and reduced localization of Swi6/HP1 and H3-Lys9-me2/me3 at the heterochromatin loci (14). A number of complexes couple RNAi with the recruitment of Clr4/Suv39 and Swi6/HP1. RNAi is involved in establishment but not in propagation of heterochromatin (15).

## UBIQUITINATION-MEDIATED ROLE OF RNAi IN SILENCING

A surprising interrelationship exists between the heterochromatin, RNAi, and ubiquitination pathways. The cullin E3 ubiquitin ligase, a component of the CLRC (Clr4-Rik1-Cul4) complex (16, 17), catalyzes monoubiquitination of Clr4/Suv39, releasing it from the ncRNA. This triggers a shift from the RNAi-dependent H3-K9-dimethylation to RNAi-independent H3-K9-trimethylation by Clr4/Suv39 (18).

The APC/C, another E3 ubiquitin ligase, also plays a role in heterochromatin assembly. *sng2-1*, a mutation in the *cut4*/*apc1* gene, encoding the largest subunit of APC/C, abrogates silencing at the *mat*, *cen*, and *tel* loci (19, 20). A direct interaction of Cut4 with Swi6/HP1 helps to assemble a repressed heterochromatin structure at the dg-dh repeats (19, 20; Fig. 1A, yellow, left panel). In contrast, mutant Cut4 fails to bind and recruit Swi6/HP1 and Clr4/Suv39, resulting in derepressed chromatin, which is associated with H3-Ac (Fig. 1a, green, right panel). Conversely, the *swi6*^−^ mutant is defective in the recruitment of Cut4 and Cut9, indicating the role of a mutually cooperative recruitment of APC/C and Swi6/HP1 and Clr4/Suv39 in heterochromatin assembly (Fig. 1A; 20). The silencing defect of *cut4* and *cut9* mutants is correlated with reduced ubiquitination activity, as it was suppressed by overexpression of the ubiquitin gene (20).

Surprisingly, similar to the *RNAi* mutants (14, 15), *cut4*, *cut9*, and *nuc2* mutants show accumulation of transcripts from forward and reverse strands of the *dh* repeats (20), indicating an additional role of APC/C in siRNA generation and heterochromatin assembly through its ubiquitination activity (Fig. 1D and d).

## NON-MENDELIAN INHERITANCE OF SILENCING DEFECT OF THE *CUT4/SNG2-1* MUTANT

However, unlike the recessive silencing mutants, like *clr3*^−^, in which the derepressed state is inherited in a Mendelian manner when crossed with a wild-type strain (2:2 segregation; Fig. 1C), the silencing defect of the *cut4^sng2-1^* mutant was dominant and segregated in a non-Mendelian manner (3:1 and 4:0 segregation; Fig. 1c) (21) and persisted, even in the wild-type segregants. Most interestingly, the derepressed state was correlated with the conversion Cut4 protein from the monomeric into multimeric form, a characteristic of prions (21, 22) (Fig. 1E). Furthermore, the derepressed state was cured by overexpression of *hsp70* and *hsp104* genes and growth in the presence of guanidine, which was accompanied by conversion of Cut4 protein from multimeric to monomeric form (21), thus confirming that the prion form of Cut4, designated as [SNG2], abrogates heterochromatin. On the contrary, like the silencing mutants (e.g., *swi6*^−^), the [SNG2] prion showed elevated rate of chromosome loss and lagging chromosomes during mitosis (21). Intriguingly, the [SNG2] prion also displayed enhanced resistance to various stresses (21) (Fig. 1F).

## FUTURE PERSPECTIVES

### Mechanism linking ubiquitination, heterochromatin assembly, and RNAi

The above results suggest that the heterochromatin proteins (along with RNAi machinery) and APC/C may function in a positive feedback loop to promote mutual recruitment and activity. Furthermore, APC/C may ubiquitinate Swi6/HP1, thereby regulating its recruitment; Swi6 may, in turn, interact with and activate APC/C. The observed suppression of the silencing defect by overexpression of the ubiquitin in the *cut4* and *cut9* mutants supports a role of ubiquitination in heterochromatin assembly. Similarly, ubiquitination may activate the RNAi factors, like Dicer, that convert the double-stranded RNAs into siRNAs to initiate heterochromatin formation (Ub; Fig. 1D). Lack of ubiquitination in *cut4* and *cut9* mutants may prevent the activation of RNAi factors, thus lowering the siRNA levels, as observed, and blocking the recruitment of Swi6/HP1 and abrogating the heterochromatin assembly (No Ub; Fig. 1d).

It is known that while polyubiquitinated proteins are degraded by the proteasome, mono-ubiquitination regulates protein function (23). Indeed, monoubiquitination of Clr4/Suv39 by the CLRC complex regulates silencing by inhibiting its property of liquid-like phase separation (LLPS) (18). Similarly, LLPS of Swi6 promotes heterochromatin assembly (24). We find that Swi6/HP1 is also monoubiquitinated by APC (unpublished data). We speculate that ubiquitination of Swi6 may regulate its function and interaction with APC/C, thus linking ubiquitination, heterochromatin formation, and cell cycle progression. This possibility is supported by the observation of lagging chromosome phenotype in *swi6*^−^ mutant (25).

### Mechanism of interference by the prion form [SNG2] in heterochromatin formation

The mechanism by which the [SNG2] prion abrogates heterochromatin structure and causes its non-Mendelian inheritance during meiosis remains unclear (21, 22). We speculate that, unlike Cut4, [SNG2] may be defective in binding and recruiting Swi6/HP1 and Clr4/Suv39, thus interfering with the heterochromatin assembly. Alternatively, lack of activation of key RNAi factors by ubiquitination in [SNG2] may prevent the generation of siRNAs and thereby the recruitment of RNAi-dependent heterochromatin proteins. [SNG2] may also affect the structure of APC and its activation by the co-activators, thus affecting normal cell cycle progression.

### Role of the [SNG2] prion form in adaptation

Most intriguingly, the prion form [SNG2] also confers resistance to various stresses, like ethanol, temperature, oxidative stress, etc. (21). This effect is especially surprising in view of the loss of heterochromatin structure and chromosome mis-segregation, indicating a defect in cell cycle progression in *cut4^−^/sng2-1* and [SNG2] strains. It is pertinent that chromosome segregation defects and enhanced stress tolerance are hallmarks of cancer (26). Notably, Cut4/Apc1 was first identified as mitotic checkpoint regulator (27). Deregulation of APC/C also affects diverse processes leading to diseases like cancer (28). More interestingly, HP1 also plays a role in cancer (29).

Mechanistically, the conversion of Cu4/Apc1 into the [SNG2] prion is correlated with the presence of intrinsically disordered regions (IDR; 21). Indeed, IDRs enhance the propensity of proteins to form prions (30). Furthermore, IDRs also endow proteins with the property of LLPS (28). Cut4/Apc1 contains at least two IDRs, which could promote both prion formation (21) and LLPS; this property may be further enhanced by the *sng2-1* mutation (21). The conformational plasticity of IDRs can contribute to altering the structure of proteins and thus help cells in adapting to fluctuating environments. Further selection can enable cells with altered protein conformations in long-term adaptation and evolution (31, 32). Paradoxically, the intrinsic plasticity can also engender the prion form, which causes chromosome instability and aneuploidy, thus leading to cancer (33). This survival mechanism may impart evolutionary advantage.

## POTENTIAL SIGNIFICANCE

A non-Mendelian pattern of inheritance of heterochromatin is not unprecedented. A repressed chromatin state of silent mating-type loci generated by overexpression of Swi6 is inherited in a non-Mendelian manner (34). Similarly, deletion of the ribonuclease Eri1 causes promiscuous spreading of heterochromatin due to increased levels of H3-K9-me and Swi6/HP1 (35). Thus, heterochromatin assembly may be governed by structure and activity of heterochromatin and RNAi factors, which are regulated through ubiquitination. Perturbation of this balance in the canonical silencing mutants as well as in the prion state of Cut4 or mutations that dysregulate APC/C or its regulators could have deleterious consequences like aneuploidy and chromosome instability and lead to cancer (32). Paradoxically, it may also confer enhanced stress tolerance and adaptability to environmental changes (21, 22).
